# Bilateral Carotid Artery Dissections and Ischemic Stroke in a Patient With COVID-19: A Case Report

**DOI:** 10.7759/cureus.31682

**Published:** 2022-11-19

**Authors:** Jessica Sop, Jordan Allen

**Affiliations:** 1 Emergency Medicine, Charleston Area Medical Center, Charleston, USA

**Keywords:** basal ganglia hemorrhage, thromboembolic event, covid-19, ais (acute ischemic stroke), non-traumatic carotid artery dissection

## Abstract

An unresponsive patient with COVID-19 infection should prompt immediate evaluation with consideration of a vast differential diagnosis entailing a multitude of diagnostic and therapeutic interventions in the emergency department. We report a case of an unresponsive 41-year-old female with COVID-19 infection and a history of rheumatoid arthritis who presented to the emergency department with bilateral carotid artery dissections and left internal carotid artery thrombus that extended into the middle cerebral artery. This case calls into question if COVID-19 is coincidentally or causally associated with acute vascular and thromboembolic disease.

## Introduction

There have been numerous reports of acute vascular and thromboembolic complications in patients diagnosed with COVID-19. Furthermore, these complications are believed to occur at a higher rate in this patient population [1]. The causal relationship between these disease processes is thought to be secondary to the inflammatory response accompanied by immune dysregulation that provokes endothelial damage and the significant coagulopathy caused by COVID-19 [2,3]. This case highlights a patient who had been diagnosed with COVID-19 and subsequently treated with antibody infusion days prior to presenting with a rare neurovascular emergency.

## Case presentation

An unresponsive 41-year-old female presented to the emergency department. The patient was unable to provide any history at the time. Emergency medical services (EMS) stated they received a call for unresponsiveness and were informed the patient was COVID-19 positive. No further history was available at the time.

**Figure 1:**
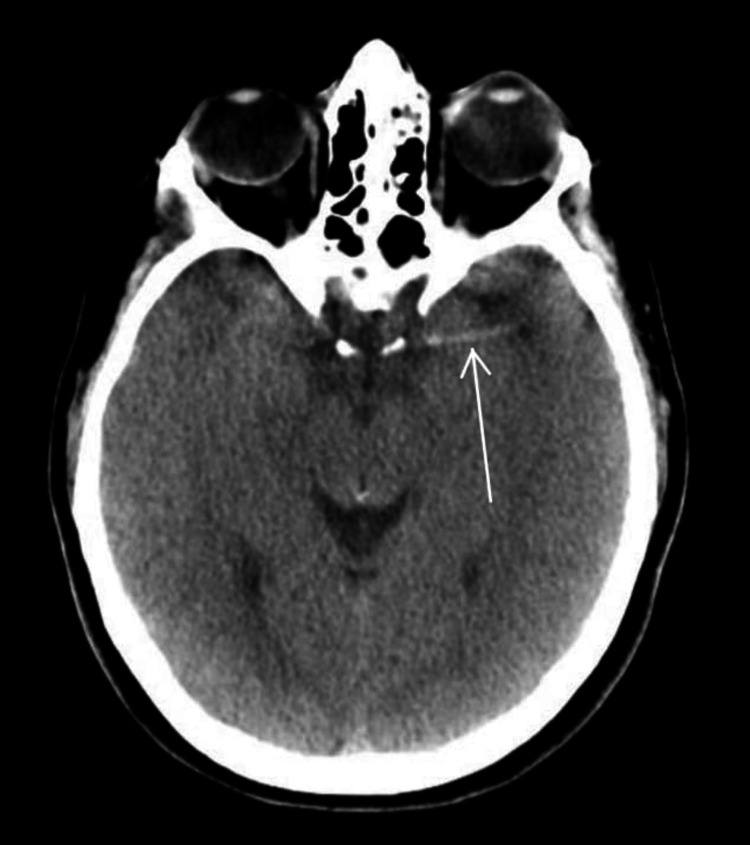
CT illustrating hyperdense MCA sign MCA - middle cerebral artery

**Figure 2:**
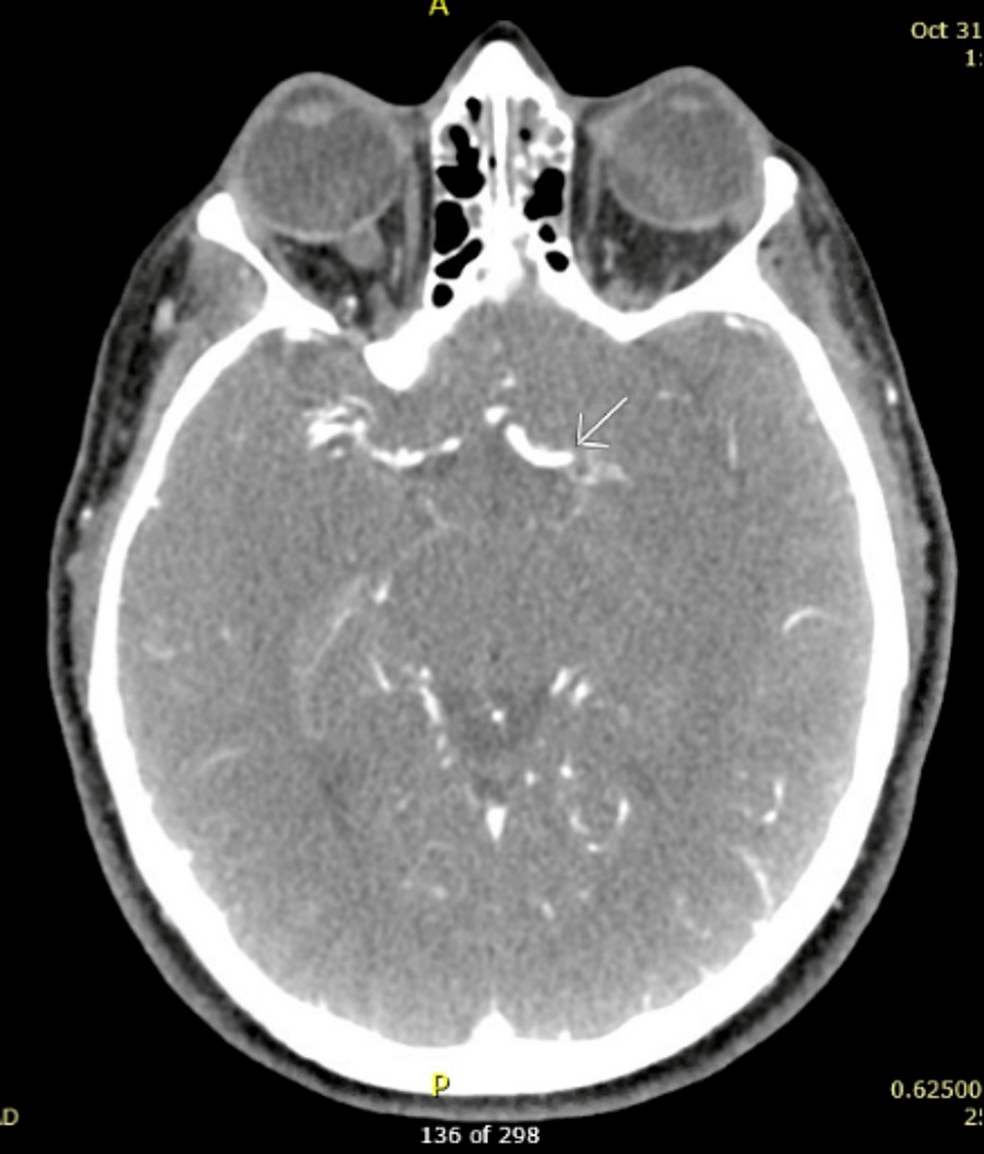
CTA illustrating left MCA occlusion CTA - computerized tomography angiogram, MCA - middle cerebral artery

**Figure 3:**
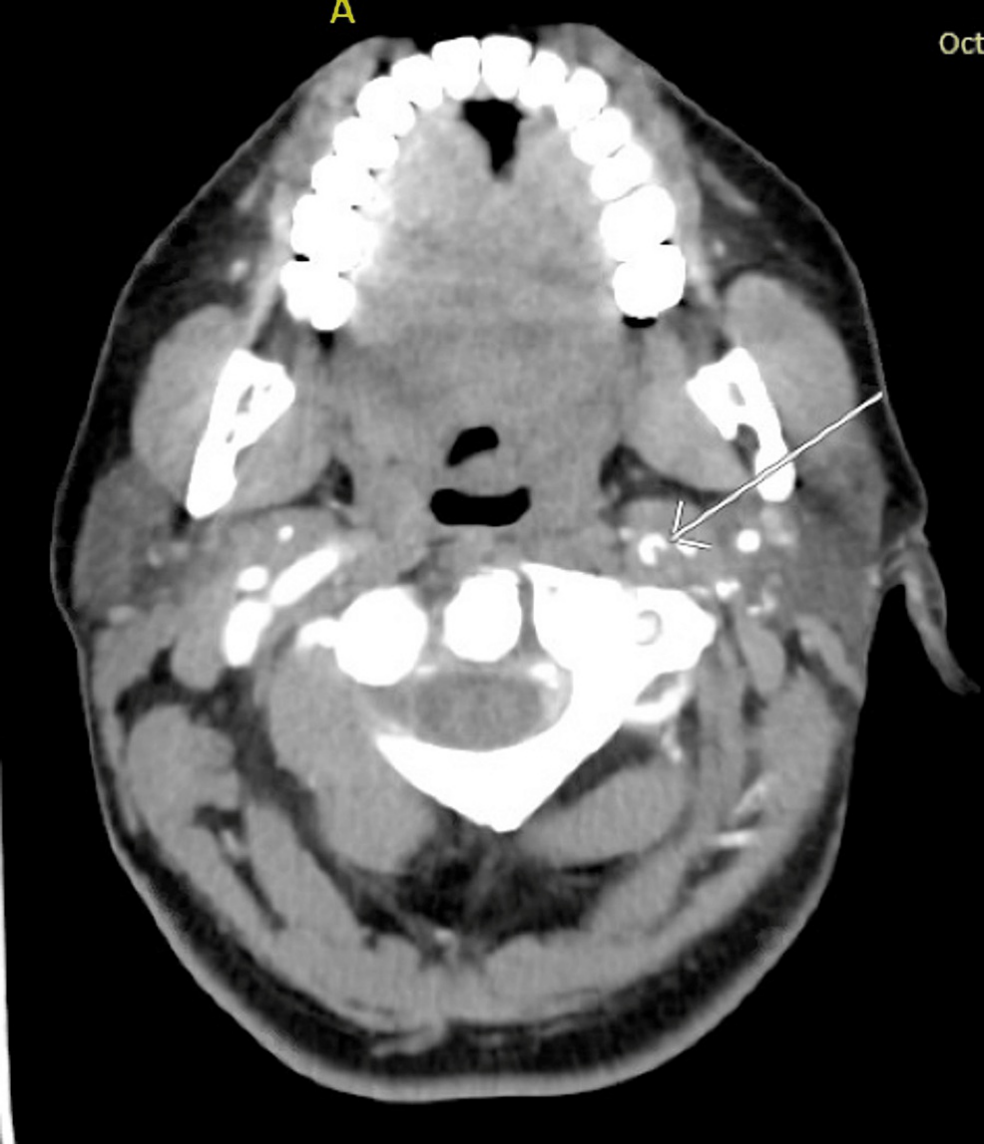
CTA illustrating left ICA dissection CTA - computerized tomography angiogram, ICA - internal carotid artery

**Figure 4:**
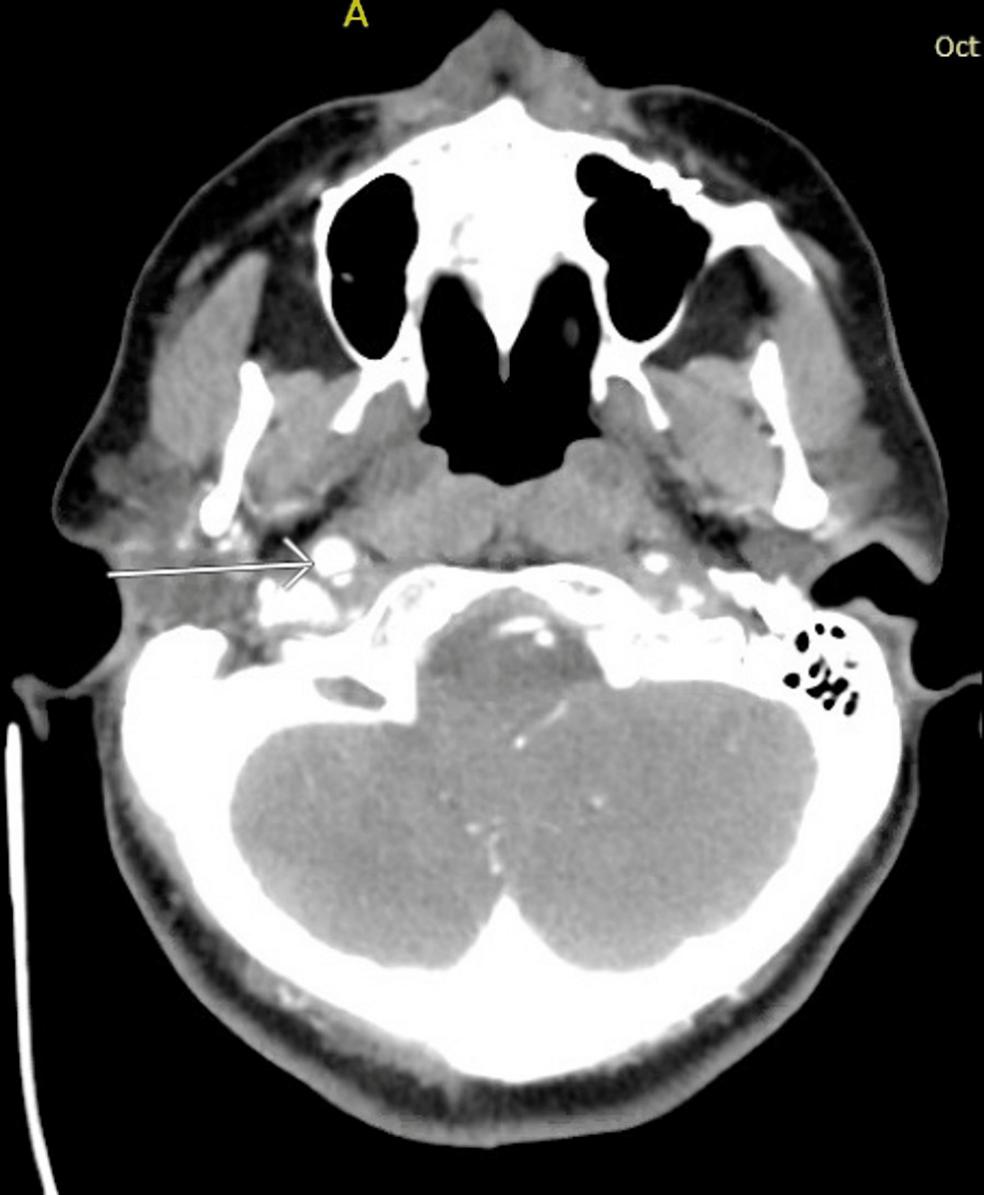
CTA illustrating right ICA CTA - computerized tomography angiogram, ICA - internal carotid artery

On presentation to the ED, the patient was awake but globally aphasic. She also had minor facial paralysis with partial gaze palsy, no movement of her right extremities, and her left extremities had no effort against gravity. Her National Institutes of Health Stroke Score (NIHSS) was 29, indicating a high likelihood of severe stroke. She was initially given 4mg of naloxone with no response. The patient expeditiously received a computed tomography (CT) of the head, which demonstrated a unique finding of a hyperdense middle cerebral artery (MCA) sign on the left with no evidence of hemorrhage (Figure 1). CT angiogram (CTA) of the head illustrated multifocal occlusions of the left intracranial internal carotid artery (ICA) and MCA with diminutive opacification throughout the left MCA territory (Figure 2). Additionally, CTA of the neck revealed concern for bilateral distal ICA dissections (Figures 3-4). The right-sided dissection did not appear to be flow-limiting; however, the left-sided dissection had focal narrowing and an intraluminal thrombus, resulting in intracranial occlusions.

**Figure 5:**
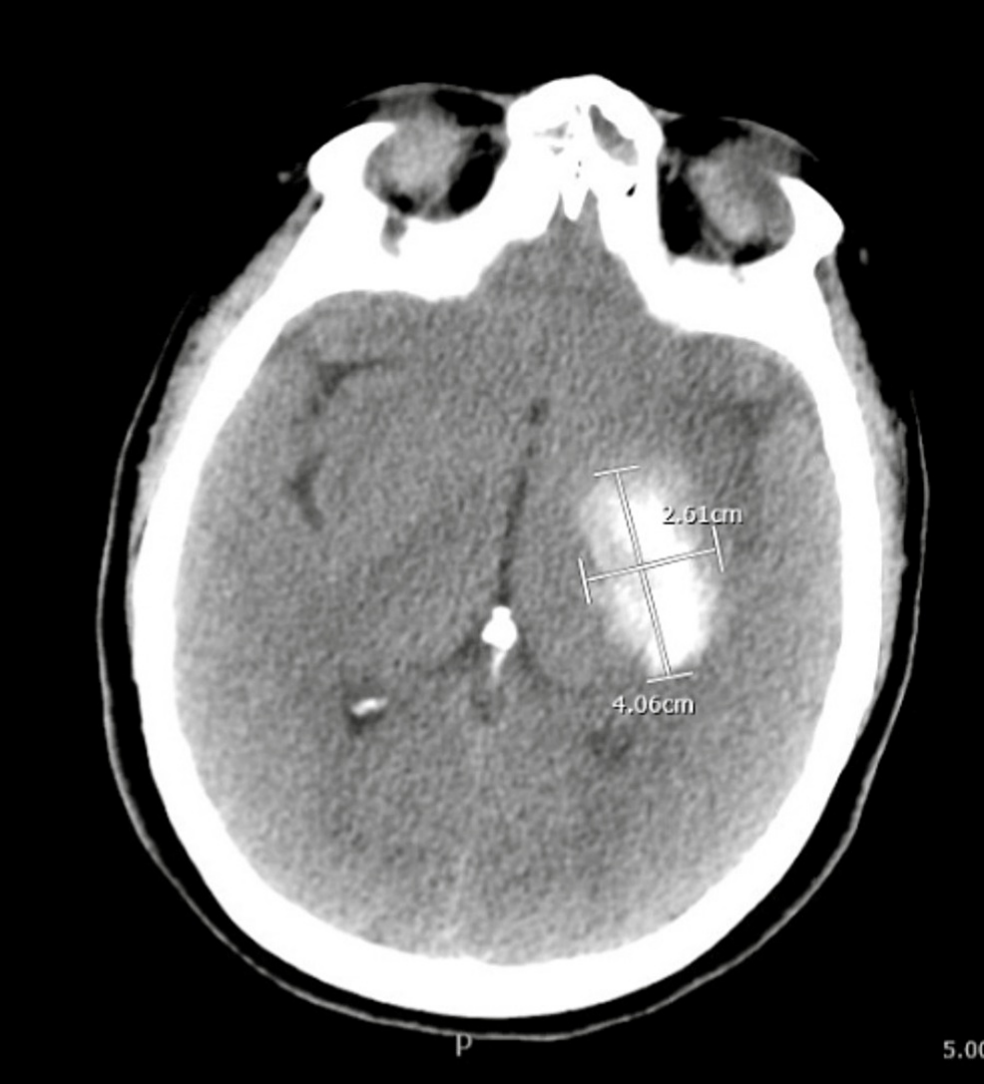
CT illustrating left basal ganglia hemorrhage

The patient was re-evaluated after returning from the CT scanner, at which time she began to develop worsening right-sided neurologic deficits. The neurology team evaluated the patient and recommended systemic thrombolysis with tissue plasminogen activator (tPA) and neurosurgical intervention. Due to worsening neurologic deficits, the patient was intubated. The neurosurgery service reviewed the patient's case and elected to perform a thrombectomy in the circulatory dynamics lab (CDL). Before leaving the emergency department for neurosurgical intervention, the patient had a tonic-clonic seizure. She consequently received a 50 mg bolus of propofol, 2g levetiracetam, and 8 mg lorazepam. The patient continued to have intermittent seizures, prompting a repeat head CT, which demonstrated no evidence of hemorrhage. The neurology service subsequently administered tPA, while the neurosurgery team retrieved a large amount of the clot burden and placed stents within the carotid arteries. A follow-up head CT performed the next morning revealed a large left-sided basal ganglia hemorrhage (Figure 5).

The patient was hospitalized for nearly one and a half months, where she steadily made a slow improvement. She continued to have residual right-sided upper and lower extremity weakness; however, she had made significant progress in her recovery. Later, it was discovered that the patient received a COVID-19 antibody infusion (casirivimab and imdevimab) the day prior to her presentation to the emergency department.

## Discussion

Carotid artery dissection can be defined as a tear within the intimal layer of the artery leading to the creation of a false lumen. Blood filling this area creates a hematoma that can eventually lead to carotid artery stenosis and thrombus formation. A thrombus may spread into the intracranial region leading to a stroke. It is also possible for the dissection to rupture, in turn leading to a subarachnoid hemorrhage [4]. Carotid artery dissection has a reported incidence of 2.6 per 100,000 per year in North America [5]. Examined historical risk factors include trauma, connective tissue disorders, infection, higher body height, lower body weight, and arterial hypertension, while other more typical vascular risk factors for stroke are not significantly associated [5,6,7]. Cervical artery dissections encompass both carotid and vertebral artery dissections. They can occur at any age; however, they are a common cause of stroke in young adults representing 20% of all strokes in this population [8].

Treatment of carotid artery dissection is multidisciplinary. Emergent consultation with neurology and interventional neurosurgical services are of the utmost importance to help guide therapy. Treatment plans will vary on a case-by-case basis, but appropriate therapeutic options could include oral medication administration, thrombolysis, or neurosurgical intervention. Medication regimens include antiplatelet therapy, systemic anticoagulation, and systemic thrombolysis if the patient has evidence of a cerebrovascular accident (CVA) and depending on the patient's causal mechanism for dissection. Interventional techniques may include mechanical thrombectomy and endovascular stenting [4]. The patient, in this case, had a significant non-traumatic large vessel occlusion and thus required systemic thrombolytics, mechanical thrombectomy, and endovascular stenting. Administration of thrombolytics is a class I treatment recommendation by the American Stroke Association and American Heart Association for the treatment of acute ischemic stroke despite the known risk of intracranial hemorrhage [9, 10]. This patient had no contraindications to the administration of tPA while having symptoms of an ischemic stroke, and as such, the standard of care was appropriately followed. The patient had significant deficits related to ischemia upon presentation; therefore, it is difficult to determine if her condition at discharge was related to the initial insult or secondary intracranial hemorrhage.

The patient, in this case, had a known history of rheumatoid arthritis, which may have contributed to her illness. In hospitalized patients, COVID-19 is thought to be an independent risk factor for stroke; and the incidence of stroke complicating COVID-19 infection is 1-6% in this population [11-13]. There have been very few reported cases in the literature of concurrent COVID-19 infection and either carotid artery dissection or bilateral carotid artery dissection. However, atypical presentations of stroke have been associated with COVID-19 [14-16]. The question remains if the patient's recent concomitant diagnosis of COVID-19 and treatment with antibody infusion had a contributing causal relationship to her stroke.

## Conclusions

This case highlights the established relationship between COVID-19 infection and thromboembolic events. In the case of COVID-19 infections, traditional risk factors for stroke and other vascular emergencies may not be applicable. The patient did receive casirivimab and imdevimab the day prior to her medical emergency. Currently, there is limited information regarding the complications of monoclonal antibodies in rheumatoid patients. Medical providers need to be aware of this in order to make appropriate diagnostic and treatment considerations regarding patients presenting with COVID-19 infections. Additional research is required to elucidate the correlation of COVID-19 to carotid artery dissection and thromboembolic events, particularly within the rheumatoid-positive population. Furthermore, this should also prompt further investigation into causal relationships between other viral illnesses and thromboembolic and vascular phenomena.
